# Identification of the *Teopod1*, *Teopod2*, and *Early Phase Change* genes in maize

**DOI:** 10.1093/g3journal/jkad179

**Published:** 2023-08-07

**Authors:** Matt Sauer, Jianfei Zhao, Meeyeon Park, Rajdeep S Khangura, Brian P Dilkes, R Scott Poethig

**Affiliations:** Department of Biology, University of Pennsylvania, Philadelphia, PA 19104, USA; Department of Biology, University of Pennsylvania, Philadelphia, PA 19104, USA; Department of Biology, University of Pennsylvania, Philadelphia, PA 19104, USA; Department of Biochemistry, Purdue University, West Lafayette, IN 47907, USA; Department of Biochemistry, Purdue University, West Lafayette, IN 47907, USA; Department of Biology, University of Pennsylvania, Philadelphia, PA 19104, USA

**Keywords:** vegetative phase change, miR156, HASTY, early phase change, EPC, Teopod, plant genetics and genomics

## Abstract

*Teopod1* (*Tp1*), *Teopod2* (*Tp2*), and *Early phase change* (*Epc*) have profound effects on the timing of vegetative phase change in maize. Gain-of-function mutations in *Tp1* and *Tp2* delay all known phase-specific vegetative traits, whereas loss-of-function mutations in *Epc* accelerate vegetative phase change and cause shoot abortion in some genetic backgrounds. Here, we show that *Tp1* and *Tp2* likely represent *cis*-acting mutations that cause the overexpression of *Zma-miR156j* and *Zma-miR156h*, respectively. *Epc* is the maize ortholog of *HASTY*, an *Arabidopsis* gene that stabilizes miRNAs and promotes their intercellular movement. Consistent with its pleiotropic phenotype and epistatic interaction with *Tp1* and *Tp2*, *epc* reduces the levels of miR156 and several other miRNAs.

## Teaser

The juvenilized phenotype of *Teopod1* (*Tp1*) and *Teopod2* (*Tp2*) mutations provided the first evidence that vegetative phase change in plants is genetically regulated. This paper demonstrates that *Tp1* and *Tp2* are hypermorhphic mutations in, respectively, *Zma-miR156j* and *Zma-miR156h*, that lead to the over-expression of miR156. Recessive alleles of Early *Phase Change* (*epc*) have the opposite phenotype, and represent mutations in the maize orthologue of *HASTY*, a gene required for the stability of miRNAs.

## Introduction

Shoot development in plants occurs in discrete phases. Upon germination, the shoot enters a juvenile vegetative phase. In most, but not all, plants, the shoot then progresses to an adult vegetative phase before entering a reproductive phase in which it produces structures involved in sexual reproduction (Poethig 2013). This process is regulated by 2 families of miRNAs, miR156/157 and miR172, which target, respectively, squamosa promoter binding/SBP-like (SBP/SPL) transcription factors and Apetala2-like (AP2-like) transcription factors (Wu and Poethig 2006; Chuck *et al*. 2007; Wu *et al*. 2009). miR156 regulates most phase-specific vegetative traits, whereas miR172 regulates phase-specific epidermal traits and flowering time (Lauter *et al*. 2005; Jung *et al*. 2007; Wu *et al*. 2009; Lian *et al*. 2021; O'Maoileidigh *et al*. 2021; Zhao *et al*. 2023).

Genetic analysis of vegetative phase change began with studies of 3 phenotypically similar dominant gain-of-function mutations in maize–*Teopod1* (*Tp1*), *Teopod2* (*Tp2*), and *Corngrass*/*Teopod3* (*Cg/Tp3*). These spontaneous mutations initially attracted interest because their phenotype strongly resembles teosinte, the ancestor of maize (Lindstrom 1925; Weatherwax 1929; Galinat 1954). This phenotype was later attributed to the prolonged expression of juvenile vegetative traits (Poethig 1988a), and subsequent analyses of *Tp1*, *Tp2*, and *Cg/Tp3* provided key insights into the nature and regulation of vegetative phase change (Poethig 1988b; Dudley and Poethig 1991, 1993; Bassiri *et al*. 1992; Evans and Poethig 1995; Bongard-Pierce *et al*. 1996). The phenotype of *Cg* is attributable to an insertion of a transposable element that causes overexpression of a transcript encoding 2 copies of miR156, *Zma-miR15b/c* (Chuck *et al*. 2007); however, the basis for the phenotype of *Tp1* and *Tp2* is still unknown. Recessive mutations that accelerate the appearance of adult traits in maize have also been identified. *glossy15* (*gl15*) causes the epidermis of juvenile leaves to resemble an adult epidermis (Evans *et al*. 1994; Moose and Sisco 1994) and is the result of a loss-of-function mutation in a AP2-like transcription factor regulated by miR172 (Moose and Sisco 1996). Consistent with this result, overexpression of miR172—which targets *Gl15*—produces an early phase change phenotype (Lauter *et al*. 2005). *early phase change* (*epc*) mutations have a pleiotropic phenotype that—depending on genetic background—ranges from seed lethality to the precocious expression of a wide range of adult vegetative traits (Vega *et al*. 2002). An analysis of natural variation in vegetative phase change in maize has also uncovered several QTLs that affect this transition (Foerster *et al*. 2015).

Here we show that *Tp1* and *Tp2* map close to and cause the overexpression of, respectively, *Zma-miR156j* and *Zma-miR156h*, strongly suggesting that they are mutations in these genes. We also demonstrate that *Epc* is the maize ortholog of the *Arabidopsis* gene, *HASTY* (*HST*).

## Materials and methods

### Plant material

Stocks of *Tp1* and *Tp2* were originally obtained from the Maize Genetics Cooperation and propagated by crossing plants heterozygous for these mutations to various inbred lines. The origin and phenotypes of *epc-W23*, *epc-1S2P*, *epc-nl4*, *epc-Mo*, and *epc-Mo* are described in Vega *et al*. (2002). *epc-3* and *epc-4* were identified in a noncomplementation screen for EMS-induced alleles. For this purpose, pollen of B73 was treated with EMS (Neuffer and Coe 1978) and crossed onto *epc-W23*/B73 plants. Two plants with a precocious “glossy” phenotype were identified in the ∼5,000 progeny of this cross, and subsequent sequencing of the *ZmHST* gene in these plants revealed the presence of point mutations in this gene. Tissue was harvested from plants grown either in the greenhouse or under field conditions.

### Nucleic acid analysis

Genomic DNA was isolated from leaf tissue using the CTAB protocol (Murray and Thompson 1980). Twelve to 15 µg of genomic DNA was separated by electrophoresis in 0.8% agarose and then transferred to Hybond N+ membranes. Hybridizations were performed in Church and Gilbert (7% SDS) buffer (Church and Gilbert 1984) with 32-P labeled, random-primed probes. Filters were visualized using a phosphoimager (Molecular Dynamics). RNA was isolated using TRIzol (GibcoBRL), and poly(A) RNA was isolated using PolyATract (Promega). Five micrograms of poly(A)-enriched RNA was separated on 1.2% agarose gels with 3% formaldehyde and transferred to Hybond N+ membranes. cDNA was made from 50 µg of total RNA using the SuperScript System for cDNA Synthesis (Life Technologies). The genomic sequence of mutant alleles of *ZmHST* alleles was determined by sequencing PCR products generated with the primers listed in Supplementary Table 1.

Northern analysis of miRNAs was conducted using 30 µg of total RNA from leaf 7 run on a 15% denaturing polyacrylamide gel containing 8 M urea. RNA was transferred to Hybond N^+^ membranes (Amersham) electrophoretically and hybridized with [γ−^32^P]-labeled probes. Oligonucleotide probes were labeled with T4 polynucleotide kinase (New England Biolabs). Hybridization was performed at 40 °C using ULTRAhyb-oligo hybridization buffer (Ambion) using probes complementary to mature miRNAs (https://www.mirbase.org). A probe to U6 snRNA (AGG GGC CAT GCT AAT CTT CTC) was used as a loading control.

The abundance of the primary transcripts of genes encoding miR156 was measured by qRT-PCR in mutant and wild-type progeny from the cross of *Tp*/W22×W22, using the primers listed in Supplementary Table 1. Total RNA was isolated from the middle of leaf 7 with TRIzol (Invitrogen), treated with Turbo DNA-Free Kit (Invitrogen) and reverse transcribed using the SuperScript III First-Strand Synthesis System (Invitrogen) with the Oligo(dT)_21_ primer. qRT-PCR was performed using the primers listed in Supplementary Table 1 in a C1000 Touch Thermo Cycler (Bio-Rad). *ACTIN* was used as endogenous control. Each data point represents 3 biological replicates, each with 3 technical replicates.

### Phylogenetic analysis

Protein sequences of exportin-like genes were collected from *Arabidopsis thaliana* genome and *Zea mays* B73 genome. The sequences were aligned with the MAFFT on XSEDE tool, and phylogeny was further determined using the RAxML-HPC on XSEDE tool on the CIPRES Gate Way (Miller *et al*. 2010).

## Results

### The identity of Tp1 and Tp2

**Fig. 1.**
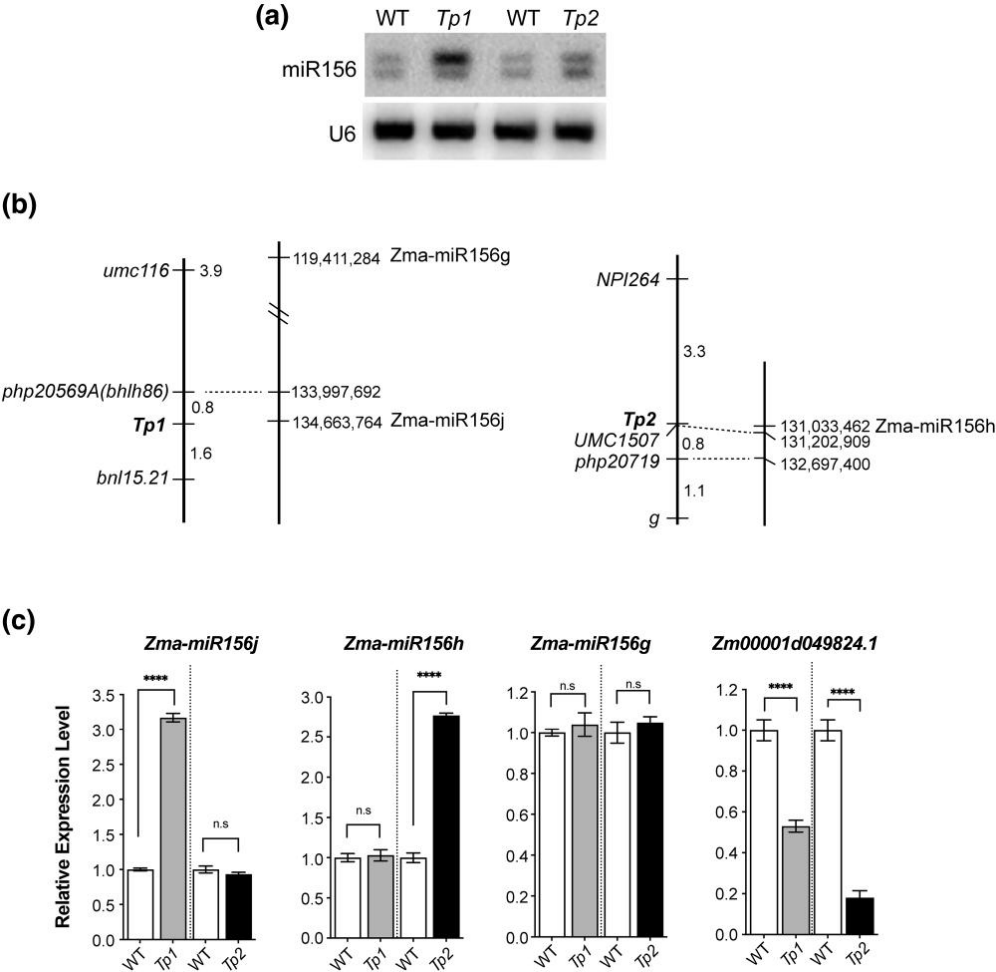
*Tp1* and *Tp2* are miR156 genes. a) Northern blot of miR156 levels in *Tp1*/+ and *Tp2*/+ and their wild-type siblings. b) Recombination (left line) and physical (right line) maps of the regions on chromosome 7 and chromosome 10 containing *Tp1* and *Tp2*. Three of the markers used for recombination mapping are located on the physical map and reveal that *Tp1* and *Tp2* are close to genes encoding miR156. c) qRT-PCR analysis of the abundance of the precursors of Zma-miR156j, Zma-miR156h, and Zma-miR156g and an SPL gene with a miR156 binding site (Zm00001d049824.1) in *Tp1*/+, *Tp2*/+, and their wild-type siblings.

Based on the phenotypic and genetic similarity between *Tp1*, *Tp2*, and *Cg*, we predicted that *Tp1* and *Tp2* were likely mutations that caused the overexpression of miR156. Northern analysis of mutant plants and their wild-type siblings confirmed this prediction (Fig. 1a). To determine the basis for the effect of these mutations on miR156 expression, we examined mapping data we had generated for *Tp1* and *Tp2* (Bongard-Pierce *et al*. 1993). Three of the markers we used have since been sequenced, making it possible to compare the position of these sites to genes encoding miR156. *Zma-miR156j* is 666 kb distal to *php20569*(*bhlh86*), which is consistent with the location of *Tp1*. *UMC1507* showed no recombination with *Tp2* and is located only 170 kb distal to *Zma-miR156h* (Fig. 1b). To determine if *Tp1* and *Tp2* affect the expression of these miR156 genes, we compared the expression of the primary transcripts of *Zma-miR156j* and *Zma-miR156h* in the 7th leaf of mutant and wild-type siblings in families segregating *Tp1* and *Tp2* (Fig. 1c). As a control, we measured the abundance of the primary transcript of *Zma-miR156g*, which is located ∼15 Mb proximal to *Zma-miR156j* on chromosome 7. Zma-miR156j was approximately 3-fold more abundant in *Tp1*/+ plants than in their wild-type siblings, whereas Zma-miR156h was ∼2.75-fold more abundant in *Tp2*/+ than in wild-type plants. *Tp1* had no effect on the expression of *Zma-miR156h*, and *Tp2* had no effect on the expression of *Zma-miR156j*, and neither mutation affected the expression of *Zma-miR156g*. To determine if the elevated level of these transcripts is functionally significant, we measured the abundance of the mRNA of the *SPL* gene, *Zm0001d49824.1*, which has a target site for miR156. This gene was repressed by about 50% in *Tp1*/+ and by more than 80% in *Tp2*/+. These results are consistent with a recent study of *Tp2* (Li *et al*. 2023) and strongly suggest that *Tp1* and *Tp2* are *cis*-acting mutations that increase the expression of, respectively, *Zma-miR156j* and *Zma-miR156h*.

### Identifying ZmHST

**Fig. 2.**
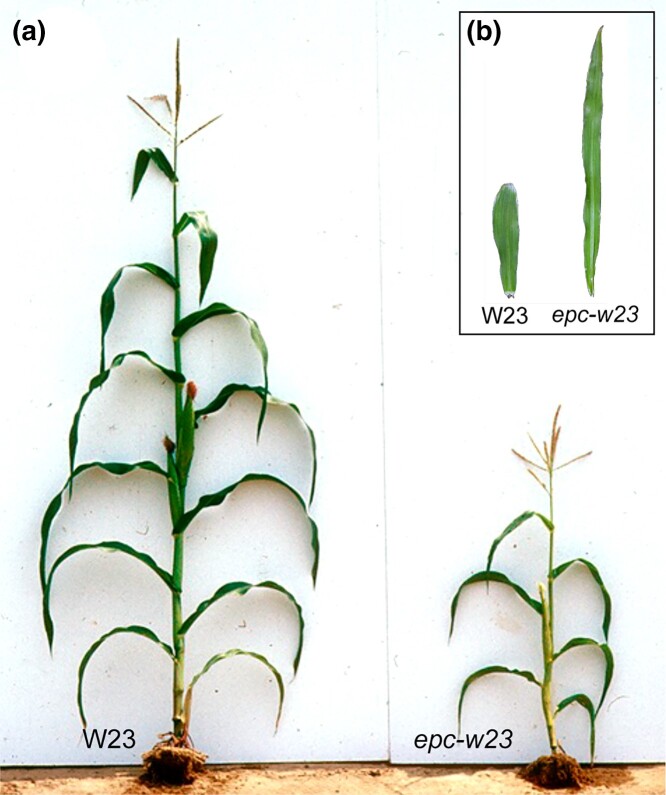
The phenotype of *epc-W23*. a) The shoot and b) first leaf of wild-type W23 and *epc-w23*.

**Fig. 3.**
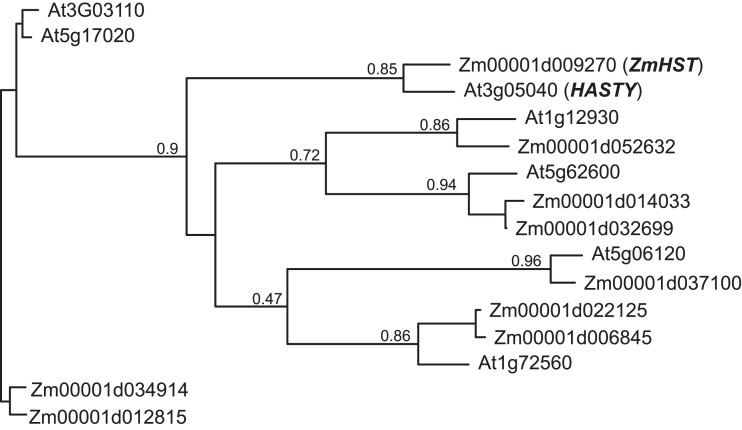
Phylogenetic tree of exportin proteins in maize (Zm) and *Arabidopsis* (At). This tree was generated using the maximum likelihood method. Bootstrap values from 1,000 replications are shown at the branch points.

**Fig. 4.**
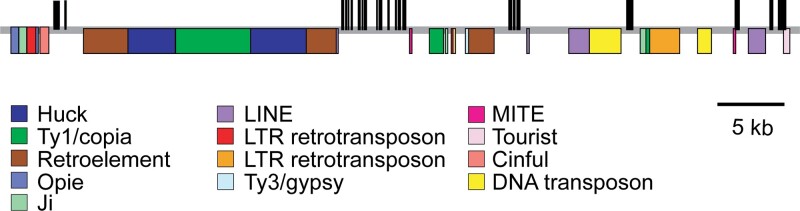
Genomic structure of *ZmHST*. Intron–exon organization of *ZmHST* and the identity of the transposable elements in introns.

In the W23 background, *epc-w23* nearly completely eliminates the juvenile phase (Vega *et al*. 2002) (Fig. 2). This phenotype is similar to that of *hst* mutants in *Arabidopsis* (Bollman *et al*. 2003), which suggested that *Epc* might be the ortholog of *HST*. Phylogenetic analysis of exportin proteins in *Arabidopsis* (Merkle 2011) and maize revealed that *HST* is most closely related to the maize gene *Zm00001d009270* (Fig. 3), hereafter referred to as *ZmHST*. *ZmHST* contains 22 exons and spans 57 kb. Its relatively large size is attributable to 4 large introns: intron 2, which is 20 kb in size, and introns 15, 18, and 19, which are all around 8 kb (Fig. 4). The majority of intron 2 consists of 3 retrotransposons inserted sequentially within one another. Introns 15, 18, and 19 also consist primarily of sequences related to various types of transposable elements.

**Fig. 5.**
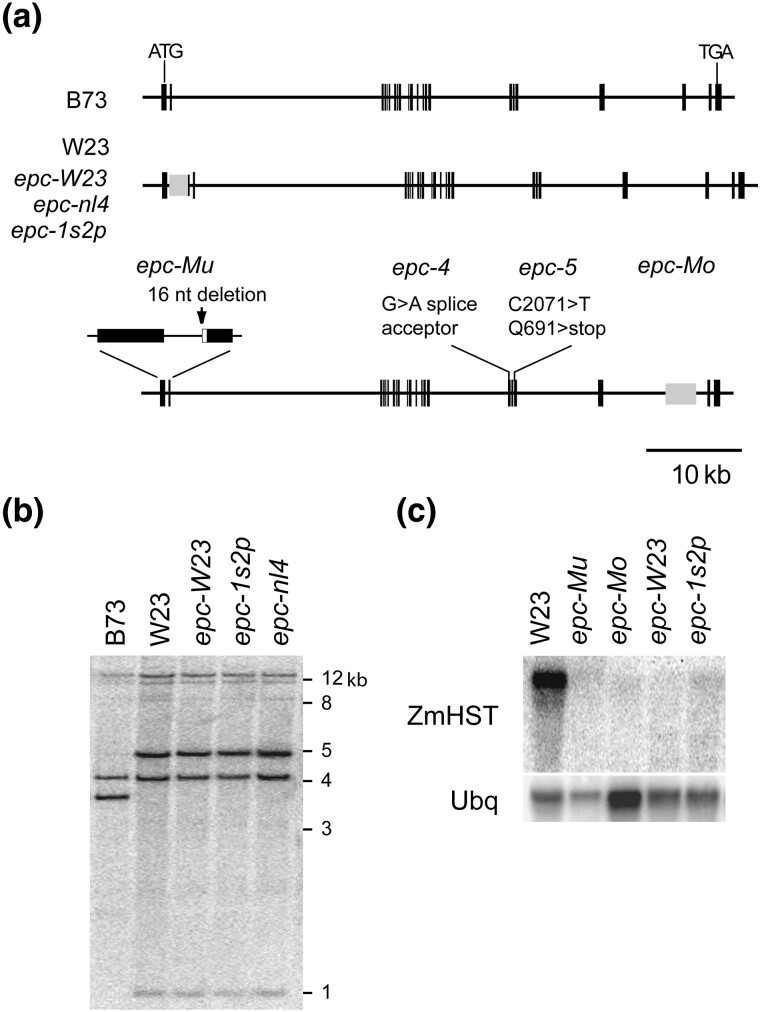
*Epc* is *ZmHST*. a) Genomic structure of *epc* alleles. b) Southern blot of HinDIII digested genomic DNA from B73, W23, and several *epc* alleles, probed with the *ZmHST* cDNA. c) Northern blot of mRNA from W23 and *epc* mutant seedlings probed with the ZmHST cDNA. Ubiquitin was used as a loading control.

To determine if *Epc* corresponds *ZmHST*, we genotyped the F2 progeny derived from the self-pollination of an Oh43/*epc-W23* plant using a probe that recognizes an EcoRV polymorphism near the 5′ end of *ZmHST*. We observed no recombination between this polymorphism and *epc-W23* in 32 mutant plants, making *ZmHST* an excellent candidate for *Epc*. We then sequenced transcripts of 4 previously described mutant alleles of *Epc* (*epc-Mu*, *epc-Mo*, *epc-W23*, and *epc-1s2p*) (Vega *et al*. 2002) (Fig. 5a). Two splice forms of *Epc* were identified in *epc-Mu.* Both contained a premature stop codon: 1 lacked exon 2, while the second was derived from a cryptic splice site in intron 1 and contained a portion of intron 1 and exon 2. Sequencing of genomic DNA from the 5′end of *epc-Mu* revealed a 16-bp deletion at the border of intron 1 and exon 2, explaining the splicing defects produced by this mutation. This deletion was absent in 15 wild-type progeny from the family in which *epc-Mu* arose, indicating that it is responsible for the mutant phenotype of *epc-Mu*. The *epc-Mo* cDNA lacked exon 20, and sequence analysis of the corresponding genomic region showed that this exon is deleted in *epc-Mo* and replaced with a non-LTR transposon. The cDNA sequences of *epc-1s2p* and *epc-W23* were identical to that of B73. However, Southern blots of these alleles revealed the presence of a ca. 1.5-kb insertion at the 5′ end of these alleles that is also present in *epc-nl4* and in the W23 inbred line in which *epc-W23* was identified (Fig. 5b). Sequencing of the corresponding genomic region from W23, *epc-W23*, *epc-1s2p*, and *nl4* demonstrated that these mutations contain an identical insertion in intron 1 consisting of a Loner retrotransposon and a duplication of a portion of exon 1 (Fig. 5a). This polymorphism is fixed in the wild-type stock of W23 that we obtained from Ed Coe, Jr, suggesting that *epc-1s2p* and *nl4* reflect the use of this W23 stock (or derivative lines) in the research programs of the investigators who identified these mutations. Two additional alleles of *Epc*—*epc-4* and *epc-5*—were identified in a noncomplementation screen using *epc-W23*/B72 as a female parent (see Materials and Methods) (Fig. 5a). The 2 alleles identified in this screen have a phenotype that is similar to that the previously described *epc* alleles (Supplementary Fig. 1).

We used Northern analysis to determine if *ZmHST* expression is altered in the mutants described above. mRNA from 3-leaf seedlings from both W23 and mutant alleles introgressed into W23 was hybridized with the *ZmHST* cDNA (Fig. 5c). All 4 of the mutants we examined had reduced levels of *ZmHST* mRNA. The observation that the *ZmHST* transcript is less abundant in *epc-W23* and *epc-1s2p* than in W23—despite the fact that W23 has the same intronic insertion as these mutants—could either mean that this Loner retrotransposon causes sporadic silencing of this locus (producing the *epc-W23* and *epc-1s2p* alleles) or that the causative polymorphism in these alleles lies in a regulatory sequence outside of the genomic region examined in our experiments.

**Fig. 6.**
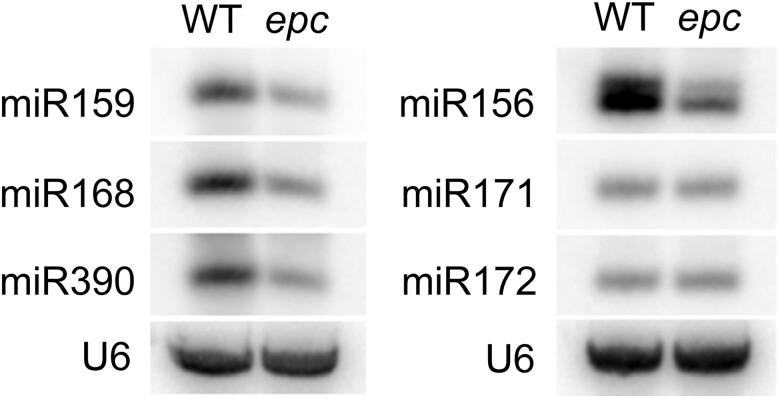
*epc-w23* has reduced levels of some miRNAs. Northern blot of small RNA from seedlings of W23 and *epc-W23* mutants, probed with oligonucleotides corresponding to various miRNAs. U6 was used as a loading control.

In *Arabidopsis*, *hst* mutations decrease the abundance of many different miRNAs (Park *et al*. 2005). To determine if this is also true for *epc*, we examined the effect of *epc-W23* on the abundance of several miRNAs by Northern analysis (Fig. 6). Four of the 6 miRNAs we examined were present at reduced levels in *epc-W23* compared to W23. This result is consistent with the morphological similarity between *epc* and *hst* and supports the conclusion that these genes have similar functions.

## Discussion

Genetic analysis of the mechanism of vegetative phase change began with the characterization of 3 dominant gain-of-function mutations–*Tp1* and *Tp2* and *Cg/Tp3*—that prolong the expression of juvenile vegetative traits (Poethig 1988a). We subsequently showed that the phenotype of *Tp1* and *Tp2* is partially suppressed by *epc-W23* (Vega *et al*. 2002), suggesting that these 3 genes are functionally related and that *Epc* acts either in parallel or downstream of *Tp1* and *Tp2*. Here we show that *Tp1* and *Tp2* cause the overexpression of, respectively, *Zma-miR156j* and *Zma-miR156h* and that *Epc* is the maize ortholog of *HST*, an *Arabidopsis* gene required for the stability and/or processing of miRNAs and their intercellular movement (Park *et al*. 2005; Zhu *et al*. 2019; Brioudes *et al*. 2021; Cambiagno *et al*. 2021). These results provide a reasonable explanation for the phenotypes and the genetic interaction between these genes.

In our original analysis of *Tp1* and *Tp2*, we used dosage analysis to determine if these dominant mutations were haplo-insufficient or gain-of-function (Poethig 1988a). For this purpose, we created plants of the genotypes, *Tp*/−, *Tp*/+, and *Tp*/+/+, using B-jA translocations. This experiment revealed that both mutations were gain-of-function but produced different predictions for the specific nature of the gain-of-function allele in each case. We and others (Li *et al*. 2023) have now shown that both mutations cause overexpression of genes encoding miR156, which raises the question of why this experiment produced different results for *Tp1* and *Tp2*. One possible explanation is that the A segments on the TB-7L and TB-10L translocations contain *SPL/SBP* genes targeted by miR156 as well as the wild-type alleles of *Tp1*(*Zma-miR156j*) and *Tp2*(Zma*-miR156h*). Specifically, *tsh4* (Zm00001eb316740) and *ZmSBP29* (Zm00001eb322280) are distal to *Tp1* on chromosome 7, and *ZmSBP21* (Zm00001eb429730) is distal to *Tp2* on chromosome 10 (Mao *et al*. 2016). All 3 of these *SPL/SBP* genes are in the same family as *AtSPL9* and *AtSPL15*, which have major but slightly different effects on vegetative phase change in *Arabidopsis* (Xu *et al*. 2016). As plants with duplications or deficiencies of 7L or 10L have altered doses of both miR156 and genes repressed by miR156, it is not surprising that the phenotypes of the *Tp*/−, *Tp*/+, and *Tp*/+/+ dosage series were not entirely consistent with the predicted effect of these genotypes on miR156 expression.

The mutant phenotype of *epc* (Vega *et al*. 2002) is similar to that of *hst* in *Arabidopsis* (Telfer and Poethig 1998) and mutations in the rice ortholog of *HST*, *CRD1* (Zhu *et al*. 2019). Like *epc*, *hst* mutations reduce the number of juvenile leaves without dramatically affecting the number of adult leaves, cause leaf curling, and reduce the growth of adventitious roots. Although the effect of *crd1* on shoot development has not been described, this mutation is similar to *epc* and *hst* in reducing adventitious (crown) root development (Zhu *et al*. 2019). In this regard, it is interesting that *ZmHST/EPC* maps within 1 cM of the maize QTLs for adventitious (brace) root development (Hostetler *et al*. 2021), which suggests that variation in the expression of *EPC* may contribute to natural variation in this trait.

One of the interesting features of *epc* is its variable expressivity. This is particularly evident in the case of *epc-W23*. In the W23 background in which it arose, the phenotype of plants homozygous for *epc-W23* can range from nearly wild type to a very strong early vegetative phase change phenotype (Vega *et al*. 2002). However, in an Oh43 or A632 background, *epc-W23* seeds fail to germinate or display shoot abortion immediately following germination. The variable phenotype of *epc-W23* in different genetic backgrounds could reflect natural variation in the expression level of the miRNAs regulated by *Epc*, differences in the expression of genes regulated by these miRNAs, or the presence of modifiers of these genes. The variable expressivity of *epc-W23* in a W23 genetic background is more difficult to explain. Northern analysis indicates that *epc-W23* is not completely null, and it may be that the processes for which it is required are hypersensitive to small variation in its activity. In *Arabidopsis*, small changes in the level of miR156 can have dramatic effects on the expression of its targets when miR156 is present at low levels (He *et al*. 2018). *epc-W23* reduces the abundance of miR156, and stochastic or environmentally induced variation in the remaining miR156 transcripts could explain the variable expressivity of *epc-W23* in a W23 background. A more interesting possibility is that the Loner retrotransposon in *epc-W23* causes stochastic, but reversible, silencing of this gene.

The evidence that mutations in *Epc* reduce the abundance of many miRNAs likely explains the pleiotropic phenotype of these mutations but also complicates efforts to determine the genes most directly responsible for the different components of this phenotype. The effect of *Epc* on vegetative phase change is likely attributable to its effect on miR156 expression, as *epc* corrects the delayed phase change phenotype of *Tp1* and *Tp2* (Vega *et al*. 2002), which cause the overexpression of miR156. The basis for the effect of *epc* on other aspects of maize development is a subject for future research.

## Supplementary Material

jkad179_Supplementary_Data

## Data Availability

Strains and plasmids are available upon request. The authors affirm that all data necessary for confirming the conclusions of the article are present within the article, figures, and tables. Supplemental material available at G3 online.
