# Correction to: Identification of the *Teopod1*, *Teopod2*, and *Early Phase Change* genes in maize

**DOI:** 10.1093/g3journal/jkad213

**Published:** 2023-09-30

This is a correction to: Matt Sauer, Jianfei Zhao, Meeyeon Park, Rajeep Khangura, Brian P Dilkes, R Scott Poethig, Identification of the *Teopod1*, *Teopod2*, and *Early Phase Change* genes in maize, *G3 Genes|Genomes|Genetics*, 2023, jkad179, https://doi.org/10.1093/g3journal/jkad179

In the originally published version of this manuscript, the fourth author’s name was incorrectly presented. His correct name is Rajdeep S. Khangura.

This error has been corrected online.

