# Supplementary material for: Identification of the Teopod1, Teopod2, and Early Phase Change genes in maize
Source: G3 (Bethesda). 2023 Aug 7;13(10):jkad179. doi: 10.1093/g3journal/jkad179 (PMC10542106; doi:10.1093/g3journal/jkad179)
Supplement: jkad179_Supplementary_Data [file jkad179_supplementary_data.zip › Table_S1_G3-2023-404450.pdf]

Table S1: PCR primers used in this study

Primer Name	Sequence (5' to 3')	Part of gene amplified
qRT-PCR of miR156 genes		
Fwd-ZmaACTIN	CCAGTGGTCGTACAACCGGTAT	
Rev-ZmaACTIN	ACCCTCGTAGATTGGCACAGT	
Fwd-Zm00001d049824	TTTGTCGAATCGGCTCTGCT	
Rev-Zm00001d049824	ACATGCCATTGATCGTTGCG	
Fwd-Zma-miR156g	GGCTGACAGAAGAGAGTGAGC	
Rev-Zma-miR156g	CTCACTTCTCTTTCTGTCAGCT	
Fwd-Zma-miR156h	TGTGCTAGTACATGCGGGAGC	
Rev-Zma-miR156h	GAGGCCCGGACCAGCGGATGAC	
Allele sequencing		
KAI-1F	TCCTGAATTTGGAAGGAGGGCGCA	exon 1
KAI-4R	GCGGAAATGCCCGCTAGTTTGACAACCA	exon 1
KAI-3F	GGACTGGCGCTCCTCCCCCGAC	exon 2
E1/E2-R	ACAAAGACAAGGACACGATTCAAGC	exon 2
E3/E6-F	TCTTGGTGGTATCCGTTAGTCCA	exon 3-6
E3/E6-R	CCCATGCGAACATGGTAAAGGTGT	exon 3-6
E7/E9-F	TTCTGTTGGGTTGCCTTGGTTCA	exon 7-9
E7/E9-R	AGCCCAAGAACAAACTAGAAGGGA	exon 7-9
E10/E12-F	TTTCACTACACAATTTCCACCCGA	exon 10-12
E10/E12-R2	ACACATTGGAGATGATGGAAGG	exon 10-12
E13/E15-F	TGGCCAGCCAAGGATTACCTTCT	exon 13-15
E13/E15-R	GGATGTGCCATAAAGCAGCTCCA	exon 13-15
E16/E18-F2	CAACTGTTTCTCTAGGGTTTCC	exon 16-18
E16/E18-R	TCTGGTGGCCATAGAAAATAAAAAGA	exon 16-18
E19-F2	GGCAGTGCCATTACGATCAG	exon 19
E19-R2	AAGCTCAAAGAATGGGAAGGTC	exon 19
E20-F	ACGTCCAGTTTTGATGGTCTCAAC	exon 20
E20-R	AGTGGTGGATTAGAGCAACCATTCA	exon 20
E21/E22-F	TGCTCTGGTACTAAATTGGAAGTGCT	exon 21-22
E21/E22-R	TCCGCCAATAATGATATGCAACCT	exon 21-22
