# Supplementary figures and images for: Identification of the Teopod1, Teopod2, and Early Phase Change genes in maize
Source: G3 (Bethesda). 2023 Aug 7;13(10):jkad179. doi: 10.1093/g3journal/jkad179 (PMC10542106; doi:10.1093/g3journal/jkad179)

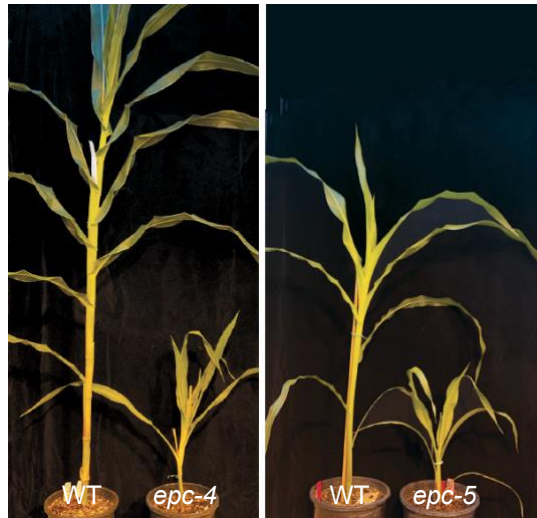

**Supplemental Figure 1: The morphology of *epc-4* and *epc-5***

Supplement: jkad179_Supplementary_Data [file jkad179_supplementary_data.zip › Figure_S1_G3-2023-404450.pdf]
